# Caffeine-induced hypokalemia: a case report

**DOI:** 10.1186/s12882-021-02465-0

**Published:** 2021-07-09

**Authors:** Min Jee Han, Su-Hyun Kim, Jung-ho Shin, Jin Ho Hwang

**Affiliations:** 1 Division of Nephrology, Department of Internal Medicine, Guro Sungsim Hospital, Seoul, Korea; 2 Division of Nephrology, Department of Internal Medicine, Chung-Ang University Hospital, 102, Heukseok-ro, Dongjak-gu, 06973 Seoul, Korea; 3 Department of Internal Medicine, Chung-Ang University College of Medicine, Seoul, Korea

**Keywords:** Coffee, Caffeine, Hypokalemia

## Abstract

**Background:**

With an increase in the global popularity of coffee, caffeine is one of the most consumed ingredients of modern times. However, the consumption of massive amounts of caffeine can lead to severe hypokalemia.

**Case presentation:**

A 29-year-old man without a specific past medical history was admitted to our hospital with recurrent episodes of sudden and severe lower-extremity weakness. Laboratory tests revealed low serum potassium concentration (2.6–2.9 mmol/L) and low urine osmolality (100–130 mOsm/kgH_2_O) in three such prior episodes. Urinary potassium/urinary creatinine ratio was 12 and 16 mmol/gCr, respectively. The patient was not under medication with laxatives, diuretics, or herbal remedies. Through an in-depth interview, we found that the patient consumed large amounts of caffeine-containing beverages daily, which included > 15 cups of coffee, soda, and various kinds of tea. After the cessation of coffee intake and concomitant intravenous potassium replacement, the symptoms rapidly resolved, and the serum potassium level normalized.

**Conclusions:**

An increased intracellular shift of potassium and increased loss of potassium in urine due to the diuretic action have been suggested to be the causes of caffeine-induced hypokalemia. In cases of recurring hypokalemia of unknown cause, high caffeine intake should be considered.

## Background

Caffeine, one of the most widely consumed drugs in daily life, is consumed by up to 90 % of the U.S. adult population [1]. While many foods contain caffeine, the majority of caffeine consumption comes from beverages such as coffee, energy drinks or shots, tea, and carbonated soft drinks [1]. The exact amount of caffeine consumed worldwide is unknown, although many countries regularly survey and report on the caffeine intake of their populations.

Overdrinking caffeinated beverages is known to cause various medical complications such as anxiety, palpitations, tremors, and gastrointestinal symptoms [2–5]. Caffeine intake can also cause electrolyte imbalance such as hypokalemia and hypophosphatemia [6, 7], but it has not been considered a major cause of hypokalemia. Here we report a case of recurrent hypokalemia induced by the heavy drinking of caffeinated beverages.

## Case presentation

A 29-year-old man without a significant past medical history visited our hospital emergency room with severe generalized muscle weakness and bilateral leg numbness that continued for several hours. He had no specific family history including diabetes mellitus and hypokalemia. His motor power values were 3 of 5 in all four extremities with normal reflexes and sensation. Blood pressure was within normal range and no other abnormalities were found on his physical examination. The patient was not under medication with herbal medicines (such as licorice), diuretics, or laxatives. No documented endocrinal dysfunction was noted. He had no symptoms such as fever, dyspnea, chest pain, palpitations, abdominal pain, or diarrhea.

His symptom onset was relatively rapid. He felt no discomfort for several years before the symptoms presented. First he felt progressive generalized muscle weakness and fatigue; after a few hours, the muscle weakness and numbness became intolerable. His laboratory examination data demonstrated considerable hypokalemia (2.6 mmol/L as K^+^), decreased serum renin (0.14 ng/mL/hr, reported 8 days after testing) and aldosterone (11.81 pg/mL, reported 8 days after testing), metabolic acidosis with respiratory compensation, and diluted urine (decreased urine osmolality, 130 mOsm/kg H_2_O) (Table 1). The transtubular potassium gradient (TTKG) was 2.43, but it was not valid because the urine osmolality was less than plasma osmolality. The spot urine potassium/creatinine ratio was 16 mEq/gCr. He was discharged from the emergency room after the muscle weakness improved by 40 mEq of intravenous potassium replacement. His potassium level was 4.4 mmol/L and he refused to be admitted for evaluation of the cause of hypokalemia. When he visited an outpatient clinic a few days later, the potassium level was within the normal range (4.3 mmol/L). Sixteen months after the first event, he visited the emergency room again with the same symptoms and signs. Serum and urine laboratory test results were similar to those of the first event: serum potassium, 2.9 mmol/L; serum renin, 0.48 ng/mL/hr; and aldosterone, 7.39 pg/mL. When the patient visited the emergency room at the first event, symptoms improved rapidly after intravenous potassium replacement and were discharged without a 24-hour urine test. In the second event, the 24-hour urine potassium excretion was measured at 57.4 mEq/24-hours while intravenous potassium replacement was performed. Two months later, a third event occurred with the same symptoms and signs. Potassium levels during the third hypokalemia event improved to 4.1 mEq/L in the next day only by recommending discontinuation of caffeine drink, without intravenous or oral potassium supplementation. As the potassium level was corrected, his generalized weakness resolved. Hypokalemia was suspected as the main cause of his muscle weakness and numbness.

**Table 1:** Laboratory biochemical test results of the patient for the 1st, 2nd, and 3rd events

	Units		Referencerange	1st event	2nd event	3rd event		At 3 days after discontinuation of caffeinated beverages	
Serum									
White blood cells	×10^6^/L		3,000–9,000	8,200	7,320	6,600		6,570	
Red blood cells	×10^12^/L		4.3–5.6	4.99	4.84	4.62		4.53	
Hematocrit	%		40–50	43.3	43.2	41.1		40.1	
Hemoglobin	g/dL		13–17	14.9	14.6	13.9		13.6	
Platelet	×10^9^/L		140–400	284	327	301		304	
Blood urea nitrogen	mg/dL		8–19	9	8	14		15	
Creatinine	mg/dL		0.67–1.17	0.70	0.68	0.69		0.82	
Creatine phosphokinase	IU/L		0-190	166	158	139			
Sodium	mEq/L		135–146	141	140	138		138	
Potassium	mEq/L		3.5–5.3	2.6	2.9	3.3		4.1	
Chloride	mEq/L		99–108	107	106	105		102	
Total CO_2_	mmol/L		21–31	22.6	24.2	28.3		29.4	
Phosphate	mg/dL		1.9–4.4	2.0	2.5	2.4		3.7	
Calcium	mg/dL		8.2–10.4	9.1	8.9	9.1		9.7	
Magnesium	mg/dL		2.0-2.5	-	2.0	2.1		2.2	
Osmolality	mOsm/kgH_2_O		280–300	293	296	-		299	
Renin	ng/mL/hr		1.31–3.95	0.14	0.48				
Aldosterone	pg/mL		30.0-355.0	11.81	7.39				
T3	ng/mL		0.60–1.81	1.20				1.45	
Free T4	mg/dL		0.89–1.76	1.17				1.39	
TSH	uIU/mL		0.55–4.78	4.03				2.21	
Arterial Blood Gas Analysis									
pH	pH		7.35–7.45		7.339	7.343			
pCO_2_	mmHg		32–48		41.1	44.2			
pO_2_	mmHg		83–108		95.7	96.1			
HCO_3_	mmol/L		22–31		21.5	23.4			
Urine									
Osmolality	mOsm/kgH_2_O		500–1200	130	111			438	
Protein	mg/dL		1–14	1.2	5.4				
Albumin	mg/L		0–30	1.9	1.9				
Creatinine (Cr)	mg/dL			17.50	27.86			58.54	
Sodium	mEq/L			38	18			100	
Potassium	mEq/L			2.8	3.4			51	
Potassium/Cr ratio	mEq/gCr			16	12.2			87.11	

Note: Conversion factors for units: serum creatinine in mg/dL to µmol/L, ×88.4; urea nitrogen in mg/dL to mmol/L, ×0.357

Hypokalemic paralysis occurred three times over 18 months. After the third episode, he agreed to be admitted and to evaluate the cause of the intermittently recurrent hypokalemia. Upon further questioning, we found that his eating habits were extremely unbalanced, and he tended to overconsume convenience and fast foods such as ramen, burger, pizza, fried chicken, and fried chips. He stated that he drank large amounts of caffeinated coffee, i.e., more than 12–20 cups a day, regardless of coffee type. His mother also mentioned that he consumed 1.5 L of Coke and fell asleep on the day of his third visit to the emergency room. Using the analytical contents of coffee, his caffeine ingestion was estimated to be > 1000 mg/day up to a maximum of 3000–4000 mg (Table 2) [1, 2, 8, 9].

**Table 2:** Caffeine content of common beverages and supplements

Item	Caffeine (mg)
Coffee [1, 7]	
Brewed from grounds, prepared with water (/8 fluid ounces)	95–333
Brewed, espresso, restaurant-prepared (/1 fluid ounce)	64
Instant, regular, powder (/8 fluid ounces)	62.7–99
Decaffeinated, brewed or instant (/8 fluid ounces)	2–35
Espresso (/1 fluid ounce)	47–64
Cola (/8 fluid ounces) [1]	24–46
Chocolate (/100 g) [6]	
100 % cocoa	240
55 % cocoa	124
33 % cocoa	45
Energy drinks (/8 fluid ounces) [1]	27–164
Caffeine contained tea (/1 serve) [1]	
Brewed black (/8 fluid ounces)	25–48
Brewed green (/8 fluid ounces)	25–29
Ready-to-drink, bottled (/8 fluid ounces)	5–40

Besides the hypokalemia, he showed abnormal liver function test results (aspartate transaminase, 48 IU/L and alanine transaminase, 102 IU/L) with a moderate degree of fatty liver, an abnormal lipid profile (total cholesterol, 255 mg/dL; triglyceride, 136 mg/dL; low-density lipoprotein, 187 mg/dL; and high-density lipoprotein, 40 mg/dL), and undiagnosed diabetes mellitus (hemoglobin A1c, 6.2 %; fasting glucose, 134 mg/dL; and post-prandial 2-hour glucose, 237 mg/dL). His body mass index was 26.0 kg/m^2^ (height 171.9 cm, body weight 76.8 kg).

Three days after he stopped the coffee and coke ingestion, his diluted urine was concentrated (from 111 to 438 mOsm/kg H_2_O), and the serum potassium level remained normal without supplementation. He was diagnosed with caffeine-induced hypokalemia. We educated him about healthy diet habits and prescribed lipid-lowering agents and metformin. After discharge, he drastically altered his lifestyle, including a reduction in his caffeine intake, regular exercise, and a decrease in his intake of convenience foods. At the last visit, an examination revealed a normal potassium level, normal liver function test results, and well-controlled diabetes mellitus and dyslipidemia.

## Discussion and conclusions

An excessive intake of caffeine might explain our patient’s clinical features. The European Food Safety Authority stated that caffeine intakes up to 400 mg/day and single doses of 200 mg do not create safety concerns for adults in the general population [10]. Our patient was reportedly ingesting > 1000 mg up to a maximum > 3000 mg of caffeine each day.

Caffeine is an effective stimulant, and many articles have been published on its toxicity following worldwide interests [4, 6, 8, 10, 11]. Although a number of case reports of caffeine-associated hypokalemia also have been reported, most of the previous reports are related to acute intake of a large quantity of caffeine and as a “one-off” [2–8, 12–15]. In one study, a caffeine ingestion of 500–600 mg/day was reported sufficient to provoke clinical symptoms (insomnia, irritability, anxiety, heart palpitations, muscle tension, heartburn, biliousness, gastritis, flatulence, and diarrhea) [13]. There was a report of significant hypokalemia after the ingestion of 180 mg of caffeine, the equivalent of 2–3 cups of regular coffee or 1–2 cups of strong coffee [9]. A regular cup of coffee has a minimum of 95 mg caffeine (range, 95–333 mg) per 8 fluid ounces [14]; however, hypokalemia has not been well-known complication of caffeine intake. In the current case, the event occurred intermittently, but repeated with a high daily intake of caffeine-containing beverages. We estimated that his potassium level was consistently lower than normal, but his symptoms and signs occurred only when his potassium level decreased to below the threshold required to maintain his muscle activity by ingesting excessive amounts of caffeine.

Hypokalemia can be induced due to a combination of the effects of caffeine and the diuretic effect of fluid drinking itself. In a recent study, the incidence of hypokalemia was significantly correlated in acute caffeine toxicity, and the higher the blood concentration of caffeine, the lower the potassium level was shown [7]. Although the detailed mechanism has not yet been clarified, losing potassium via the urine stream due to the diuretic action of caffeine is proposed as one possibility [16, 17]. In addition, caffeine promotes diuresis and natriuresis by competitively binding to adenosine receptors A1 and A2_A_, and the lifestyle of high caffeine consumption while on a high sodium diet like this patient may be associated with more potent polyuria and subsequent potassium loss [10]. Caffeine induces potassium redistribution into the cells and increases renal losses by inhibiting phosphodiesterase, which increases cyclic adenosine monophosphate (cAMP) levels. Increased cAMP activates sodium-potassium adenosine triphosphates (ATP), causing a shift of extracellular potassium into the intracellular compartment [18]. It has been known that caffeine also reduces the conductance of potassium-ATP channels, but contradicting data exist on whether it mainly acts as an inhibitory or stimulatory effect on the channel [19–21]. In another potential mechanism, caffeine stimulates the beta-adrenergic system and increases renin release [12]. When the renin-angiotensin-aldosterone system is activated, potassium loss increases. However, the patient in this case showed constant low plasma renin activity and aldosterone level in repeated tests. In general, caffeine is associated with renin-angiotensin-aldosterone system activation as an acute effect especially in subjects who have not been exposed to caffeine, but patients with chronic caffeine consumption have reported lower renin levels than normal [22]. Severe hypokalemia also suppresses aldosterone secretion [23]. He seldom drink still water, and mostly hydrated with coffee or soda. In addition to the effect of caffeine, a large amount of fluid intake itself may have intensified hypokalemia by increasing flow-induced potassium secretion through the large conductance Ca^2+−^activated potassium (BK) channel [24]. Caffeine-induced elevated cardiac output increases the glomerular filtration rate, which induces caffeine-related diuresis and subsequent potassium excretion [15, 17]. Although it is a relatively minor effect, caffeine also activates the respiratory center of the brain, causing hyperventilation, and the resultant respiratory alkalosis possibly induces hypokalemia [16]. In this patient, metabolic acidosis with respiratory compensation was observed, which is also reported as a part of acute toxicity of caffeine [11].

This patient had no history of diabetes, but was diagnosed with diabetes mellitus through serially measured blood sugar and hemoglobin A1c after hospitalization as the third event. Hypokalemia has been reported to be associated with diabetes mellitus in various ways. Hypokalemia is known to be associated with impaired insulin secretion by hypokalemia-mediated beta-cell dysfunction, increased hepatic glucose production, and decreased peripheral glucose utilization resulting in glucose intolerance and hyperglycemia [25, 26]. Also, there is a study showing that insulin sensitivity was restored after correction of hypokalemia [27]. In this patient, chronic clinical or subclinical hypokalemia may have been associated with diabetes mellitus. However, although the hypokalemia was completely improved and did not recur through the change of the diet (mainly on reducing caffeine), the oral anti-diabetic drug was not able to stop, so it is considered that his diabetes was a part of metabolic syndrome.

At our patient’s first and second visits, we tried to calculate the TTKG from his spot urine values, but there was a limitation to interpreting the TTKG because his urine osmolality was lower than his serum osmolality. He reported urinating at least 10 times a day while awake, and our findings suggested that his polyuria was related to the excessive caffeine intake itself and urinary concentrating defect in hypokalemia [28]. That also explained why his spot urine osmolality was too diluted to calculate TTKG. In this patient, 24-hour urine potassium excretion was measured once and high potassium excretion was shown. However, since clinically urgent potassium repletion was required at the time, the urine collection was proceeded with concomitant IV potassium replacement. Therefore, although the 24-hour urine potassium excretion is the most accurate method to measure urinary potassium excretion, it is thought that potassium excretion measured by spot urine is more meaningful than the value of 24-hours potassium excretion in this setting.

Excessive caffeine intake was not considered as a major cause of hypokalemia in previous studies. Although there are individual differences, modern people ingest reasonable amounts of caffeinated beverages daily. However, physicians should pay attention to patients’ drinking habits of caffeinated beverages during evaluations of hypokalemia.

In conclusion, excessive caffeine intake can cause repetitive episodes of hypokalemia. Physicians should pay more attention to patients’ caffeine intake as well as several metabolic or endocrinological causes during the work-up for hypokalemia by taking a detailed history.

## Data Availability

Records and data pertaining to this case are in the patient’s secure medical records in the Chung-Ang University Hospital. If needed, the relevant material can be provided by corresponding author on reasonable request.

## Abbreviations

cAMP: Cyclic adenosine monophosphate
TTKG: Transtubular potassium gradient

## References

[1] Mitchell DC, Knight CA, Hockenberry J, Teplansky R, Hartman TJ. Beverage caffeine intakes in the U. S. Food Chem Toxicol. 2014;63:136–42. 10.1016/j.fct.2013.10.042 24189158

[2] Alazami M, Lin SH, Cheng CJ, Davids MR, Halperin ML (2006). Unusual causes of hypokalaemia and paralysis. QJM: monthly journal of the Association of Physicians.

[3] Aizaki T, Osaka M, Hara H, Kurokawa S, Matsuyama K, Aoyama N, Soma K, Ohwada T, Izumi T (1999). Hypokalemia with syncope caused by habitual drinking of oolong tea. Internal medicine.

[4] Abbott PJ (1986). Caffeine: a toxicological overview. The Medical journal of Australia.

[5] Appel CC, Myles TD (2001). Caffeine-induced hypokalemic paralysis in pregnancy. Obstetrics gynecology.

[6] Kamijo Y, Takai M, Fujita Y, Usui K (2018). A Retrospective Study on the Epidemiological and Clinical Features of Emergency Patients with Large or Massive Consumption of Caffeinated Supplements or Energy Drinks in Japan. Internal medicine.

[7] Tsuji T, Morita S, Saito T, Nakagawa Y, Inokuchi S (2020). Serum potassium level as a biomarker for acute caffeine poisoning. Acute Med Surg.

[8] Temple JL, Bernard C, Lipshultz SE, Czachor JD, Westphal JA, Mestre MA (2017). The Safety of Ingested Caffeine: A Comprehensive Review. Front Psychiatry.

[9] Gilbert RM, Marshman JA, Schwieder M, Berg R (1976). Caffeine content of beverages as consumed. Canadian Medical Association journal.

[10] The European Food Safety Authority Panel on Dietetic Products NaA (2015). Scientific Opinion on the safety of caffeine. EFSA Journal.

[11] Willson C (2018). The clinical toxicology of caffeine: A review and case study. Toxicol Rep.

[12] Gennari FJ (1998). Hypokalemia. N Engl J Med.

[13] Packer CD (2008). Chronic hypokalemia due to excessive cola consumption: a case report. Cases J.

[14] Passmore AP, Kondowe GB, Johnston GD (1986). Caffeine and hypokalemia. Ann Intern Med.

[15] Tajima Y (2010). Coffee-induced Hypokalaemia. Clin Med Insights Case Rep.

[16] Benowitz NL. Clinical pharmacology of caffeine. Annual review of medicine 1990, 41:277–288. 10.1146/annurev.me.41.020190.001425 2184730

[17] Arnaud MJ (1987). The pharmacology of caffeine. Progress in drug research Fortschritte der Arzneimittelforschung Progres des recherches pharmaceutiques.

[18] Bradberry SM, Vale JA (1995). Disturbances of potassium homeostasis in poisoning. J Toxicol Clin Toxicol.

[19] Mao X, Chai Y, Lin YF (2007). Dual regulation of the ATP-sensitive potassium channel by caffeine. Am J Physiol Cell Physiol.

[20] Chorvatova A, Hussain M (2003). Effects of caffeine on potassium currents in isolated rat ventricular myocytes. Pflugers Arch.

[21] Varro A, Hester S, Papp JG (1993). Caffeine-induced decreases in the inward rectifier potassium and the inward calcium currents in rat ventricular myocytes. Br J Pharmacol.

[22] Palatini P, Canali C, Graniero GR, Rossi G, de Toni R, Santonastaso M, dal Follo M, Zanata G, Ferrarese E, Mormino P (1996). Relationship of plasma renin activity with caffeine intake and physical training in mild hypertensive men. HARVEST Study Group. Eur J Epidemiol.

[23] Himathongkam T, Dluhy RG, Williams GH (1975). Potassim-aldosterone-renin interrelationships. J Clin Endocrinol Metab.

[24] Rieg T, Vallon V, Sausbier M, Sausbier U, Kaissling B, Ruth P, Osswald H (2007). The role of the BK channel in potassium homeostasis and flow-induced renal potassium excretion. Kidney Int.

[25] Liamis G, Liberopoulos E, Barkas F, Elisaf M (2014). Diabetes mellitus and electrolyte disorders. World J Clin Cases.

[26] Eriksson JW, Jansson PA, Carlberg B, Hagg A, Kurland L, Svensson MK, Ahlstrom H, Strom C, Lonn L, Ojbrandt K (2008). Hydrochlorothiazide, but not Candesartan, aggravates insulin resistance and causes visceral and hepatic fat accumulation: the mechanisms for the diabetes preventing effect of Candesartan (MEDICA) Study. Hypertension.

[27] Tourniaire J, Bajard L, Harfouch M, Rebattu B, Garrel D (1988). Restoration of insulin sensitivity after correction of hypokalemia due to chronic tubulopathy in a diabetic patient. Diabete Metab.

[28] Amlal H, Krane CM, Chen Q, Soleimani M (2000). Early polyuria and urinary concentrating defect in potassium deprivation. Am J Physiol Renal Physiol.

